# LoMuS: low-rank adaptation with sequence multi-representation improves protein stability prediction

**DOI:** 10.1093/bioinformatics/btag509

**Published:** 2026-07-09

**Authors:** Samuel Infante, Akash Singh, Anowarul Kabir

**Affiliations:** Bellini College of AI, Cybersecurity and Computing, University of South Florida, Tampa, Florida 33620, United States; Bellini College of AI, Cybersecurity and Computing, University of South Florida, Tampa, Florida 33620, United States; Bellini College of AI, Cybersecurity and Computing, University of South Florida, Tampa, Florida 33620, United States

## Abstract

**Motivation:**

Protein folding stability is a key determinant for understanding protein dynamics, including molecular function, pathogenicity, and protein engineering. Yet, accurate prediction of protein stability remains challenging due to high variability in available data, particularly when only sequence information is available and structural knowledge is limited or unavailable. In this work, we introduce LoMuS, a multi-representation-based deep learning model that predicts dataset-provided protein stability scores directly from the primary sequence. In the core of the model architecture, a fusion network integrates explicit physicochemical descriptors with low-rank adapted protein language model derived embeddings from the sequence that consistently gains across standard experimental stability benchmarks.

**Results:**

We rigorously evaluate LoMuS across multiple settings, such as absolute folding stability scoring, mutation landscape stability scoring, held-out protein domains, out-of-distribution label regimes, and per-protein evaluation. LoMuS consistently outperforms sequence-only baselines, achieving an absolute performance gain of at least 10% in Spearman’s rank correlation across several benchmarks. Per-protein evaluations further demonstrate robust performance gains. Ablation analyses confirm that complementary signals from physicochemical descriptors and sequence embeddings are critical to the effectiveness of the proposed multi-representation approach. We believe LoMuS advances protein engineering research by improving the prediction and ranking of protein stability scores.

**Availability:**

All codes including data preparation scripts, training and validation recipes, and experimental configurations for LoMuS are available at: https://github.com/kabir-ai2bio-lab/LoMuS.

## Graphical Abstract

**Graphical Abstract:**
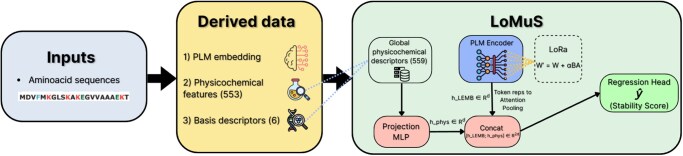

## 1 Introduction

Reliable protein stability prediction has the potential to transform biotechnology, human health, drug repurposing and medical therapeutics. Stable proteins are often used as enzymes in biopharmaceuticals and safe industrial food (Masson and Lushchekina 2022). Conversely, destabilizing missense mutations represent a primary mechanism of pathogenicity in diseases such as Alzheimer’s, cystic fibrosis, and Lynch syndrome (Clausen *et al.* 2019, Blaabjerg *et al.* 2023, Cheng *et al.* 2023), since many pathogenic variants act by lowering protein stability (Savojardo *et al.* 2024). By elucidating the core in the sequence-structure-function relationship, accurate prediction of protein folding stability can aid in rational design in protein engineering and variant interpretation (Alley *et al.* 2019).

Protein stability generally refers to the free-energy gap between native ($G_{N}$) and unfolded ($G_{U}$) ensembles under certain conditions, defined as $\Delta G_{\mathrm{fold}}=G_{N}-G_{U}$. This folding stability can also be changed due to mutations, measured as $\Delta \Delta G=\Delta G_{\mathrm{fold}}^{MT}-\Delta G_{\mathrm{fold}}^{WT}$ where $\Delta G_{\mathrm{fold}}^{MT}$ and $\Delta G_{\mathrm{fold}}^{WT}$ indicates free-energy gap for mutant- and wild-type variants of the same protein molecule, respectively. Here, depending on the ΔΔG equation, positive and negative values denote destabilization and stabilization, respectively. Experimental ΔG and ΔΔG are typically derived under two-state assumptions from chemical denaturation or thermal unfolding and mapped to free-energy with standard analyses (Hamborg *et al.* 2020, Seelig and Seelig 2023). Being low-throughput, laborious, and expensive, such methods are not widely applicable across many proteins or large-scale variants screening. To address this problem, several computational methods have been proposed over the years; however, they often suffer from scaling and generalizability due to the out-of-distribution problem (Shi *et al.* 2025), often raised within biological domain.

Classical methods are computationally expensive and limited in scale due to the usage of empirical energy functions based on physical chemistry, such as FoldX (Guerois *et al.* 2002) and Rosetta (Kellogg *et al.* 2011). They compute ΔΔG from changes in molecular energy using force fields and free-energy centric simulations, makes them unamenable even for a modest number of variants (Sanavia *et al.* 2020). Additionally, methods relying on protein three-dimensional structures are not applicable when such structures are not available (Laimer *et al.* 2015). Consequently, classical models struggle with large-scale mutational scanning and often not usable for long proteins.

Machine learning (ML) approaches train statistical methods on existing stability data. mCSM (Pires *et al.* 2014) models the residue environment using graph-based signals to predict stability changes. DeepDDG (Cao *et al.* 2019), an early neural-network model, uses mutation centric residue features trained on approximately 5700 mutations to predict mutation-induced stability. Ensemble methods, i.e. MAESTRO (Laimer *et al.* 2015), combine multiple regression agents, and newer tools, i.e. PremPS (Chen *et al.* 2020), employ random forests with structure- and evolution-based features. Most ML models rely on limited curated ΔΔG datasets, and high-quality structures for feature extraction. They often tend to overfit common protein families and offer only modest accuracy for novel cases. DeepDDG, for instance, achieved pearson’s correlations of only 0.5–0.6 on independent test sets (Cao *et al.* 2019). Many predictors also compress ΔΔG estimates toward zero, underestimating large stability shifts and often missing strongly stabilizing mutations (Pancotti *et al.* 2022). While some methods try to counter this via dataset balancing, their accuracy can still degrade when structural inputs are low-quality (Chen *et al.* 2020).

Although data-driven methods have improved folding stability prediction, recent deep learning-based advancements still inherit key constraints. CNNs on structural micro-environments [RaSP (Chu *et al.* 2024)] and graph-based models that fuse sequence embeddings with 3D information [ELASPIC-2 (Strokach *et al.* 2021)] report improved performance. PMSPcnn (Sun *et al.* 2024) uses persistent homology extracted topological features to predict stability effect caused by point mutations and improves over prior baselines. Hybrid designs continue this trend, for example DDMut (Zhou *et al.* 2023), a siamese network that integrates graph encodings of local 3D context with convolutional and transformer layers, and ProS-GNN (Wang *et al.* 2023) uses message passing on atomistic graphs to capture both short- and long-range effects. Because these methods require structural data and curated labels, they are less applicable when structures are missing or low-resolution. This motivates sequence-first models augmented with readily available biochemical features.

Recent trend leverages protein language models (PLMs) pretrained on a large corpus of protein sequences (Rao *et al.* 2019, Elnaggar *et al.* 2022, Lin *et al.* 2023). They provide powerful general-purpose embeddings that can be transferred into diverse relevant prediction tasks, including secondary structure, disorder, solvent accessibility, mutation effect, function and stability (Kabir and Shehu 2022, Chandra *et al.* 2023, Bromberg *et al.* 2024). Crucially, Schmirler *et al.* (Schmirler *et al.* 2024) showed that supervised fine-tuning of PLMs almost always improves downstream protein prediction tasks, however might subject to overfitting (Kabir *et al.* 2024). Transformer PLMs can capture implicit structural information and enable accurate tertiary structure prediction from a single sequence without MSAs (Fang *et al.* 2023). Another method, augmented with structural or physicochemical inputs combined with PLM embeddings, reported improved performance for protein stability change (ΔΔG) estimation (Zhang *et al.* 2023). Generative and task-conditioned LLMs also support protein engineering workflows; a temperature-guided model achieved more than 30% experimental success in proposing stabilizing mutations across multiple proteins (Jiang *et al.* 2023). Another class of parameter-efficient methods such as low-rank adapters (LoRA) can achieve on-par performance while cutting trainable parameters (Hu *et al.* 2021). Overall, these results suggest that careful task-specific adaptation can lead to capturing subtle stability changes caused by mutation. Very recently, multimodal protein representation methods such as ProSST (Li *et al.* 2024) and ProMEP (Cheng *et al.* 2024) explicitly incorporate 3D structural context (e.g. structure tokenization or structure embeddings) alongside sequence, which can improve performance in settings where reliable structures are available.

Considering the drawbacks and progress discussed earlier, we propose a deep learning based computational approach that considers only protein sequence as input, no dependency on structures or MSAs, and leverages a multi-representation architecture designed to improve performance across standard stability benchmark settings. Here, we introduce LoMuS, a low-rank adaptation of embeddings with multi-representation protein features to predict protein stability scores, including absolute folding stability (ΔG) and mutation landscape assay scores, depending on the dataset. LoMuS consists of three core components, (i) global physicochemical features that we derive from the given sequence, (ii) a LoRA adapted PLM that we finetune in-house by freezing most weights and only learning small rank-decomposition adapters in each Transformer layer (Hu *et al.* 2021), and (iii) a fusion network where PLM embeddings and the global representation are fused and passed through a lightweight neural head. Intuitively, the model learns to the contribution of the learned sequence embedding versus the physicochemical features. This design avoids the additional computational and storage overhead of generating or processing structures at scale and reduces sensitivity to structural noise or uncertainty in predicted structures, especially when evaluating very large mutational landscapes.

We train LoMuS on experimental stability datasets and report performance under evaluation settings commonly used in prior works, including domain-heldout splits, label-shift regimes, and per-protein DMS evaluations. The resulting model combines LoRA-adapted sequence embeddings with compact physicochemical descriptors and achieves strong performance across stability benchmarks. Across held-out domains and out-of-distribution stability label regimes, LoMuS improves Spearman’s rank correlation by roughly 10–13% over strong sequence-based baselines. We further evaluate LoMuS on a broader set of 66 ProteinGym DMS assays of 53 unique proteins used by ProMEP, which include heterogeneous mutation-effect readouts beyond stability, such as binding, activity, fluorescence, and fitness. Although this benchmark is not the primary target setting for LoMuS and differs from ProMEP’s zero-shot structure-informed protocol, it provides an additional test of whether the learned sequence and physicochemical representations transfer beyond stability-only labels. Overall, these results suggest that fusing a LoRA-adapted PLM with lightweight biochemical signals improves stability ranking across domains while also showing promising per-protein generalization on broader mutational scanning tasks.

In the rest of the article, we discuss our methodological contributions and subsequently we report and compare our results with the state-of-the-art methods. Section 2 presents LoMuS, including the LoRA fine tuning strategy, physicochemical feature set, fusion network, and training setup. We then summarize the datasets and preprocessing steps in Section 3. Section 5 reports results with standard performance metrics and ablation studies to understand various model choices.

## 2 Methodology

### 2.1 LoMuS overview

**Figure 1:**
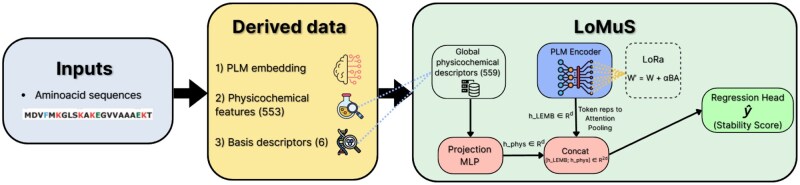
Procedural overview of the LoMuS architecture. Given an amino acid sequence as input, LoMuS derives three sets of features: (i) embeddings generated from a PLM, (ii) physicochemical descriptors, and (iii) basis features, including sequence length, molecular weight, aliphatic index, instability index, and related global descriptors. The sequence is encoded by a pretrained PLM and adapted with LoRA on the attention projections. In parallel, the physicochemical feature vector is standardized and mapped by a multi-layer perceptron (MLP) into the same PLM feature dimension. The model concatenates both representations and feeds them to a lightweight regression head that outputs a stability score. The target is the dataset-provided stability label, such as absolute folding free energy (ΔG) when reported, or an assay-derived score otherwise. This fusion lets the network weight information from the learned sequence embedding and the explicit physicochemical context during end-to-end training.

We propose a multi-representation deep learning architecture, namely LoMuS, that estimates the stability score of a protein molecule from the primary sequence as input. Our model integrates three sets of features: (1) sequence embeddings generated by a pretrained PLM, (2) physicochemical descriptors and (3) basis features, summarized in Fig. 1. We leverage the ESM-2 transformer-based encoder (Lin *et al.* 2023) and fine-tune it using LoRA (Hu *et al.* 2021) while preserving most pretrained weights to extract sequence embeddings. The physicochemical features are derived from AAindex (Nakai *et al.* 1988, Tomii and Kanehisa 1996, Kawashima *et al.* 1999, Kawashima and Kanehisa 2000). Subsequently, we compute six basis descriptors of the input molecule. Finally, our designed fusion network combines all of those features and accurately predict the stability scores. The following subsections describe each component of our architecture in detail.

### 2.2 From input sequence to learned representation

We use the ESM-2 (Lin *et al.* 2023) (650 million parameters) to extract the learned representation of the input protein sequence. ESM-2 is pretrained on the UniRef50 (Suzek *et al.* 2015) corpus comprising billions of protein sequences. With multiple self-attention layers, it is designed to capture implicit patterns in sequences correlated with underlying structure and function. Each sequence is then fed into the ESM-2 encoder to produce a contextual embedding for each residue. Given an input amino acid sequence of length *L*, ESM-2 (hidden size d=1280) yields embeddings $X\in R^{L\times d}$.

### 2.3 Learning task-relevant features using low-rank adaptation fine-tuning

To adapt the PLM to our task-of-interest, stability prediction, we apply LoRA (Hu *et al.* 2021) to the model’s attention layers. LoRA is a parameter-efficient fine-tuning technique (PEFT) that freezes the original pretrained weights and injects trainable low-dimensional matrices into each layer’s weights. This reduces the overfitting risk under limited labeled data and retains PLM knowledge for improving out-of-distribution generalization performance (Liu *et al.* 2023), as in ours.

In practice, for each attention projection weight matrix *W* in the PLM (query *Q*, key *K*, value *V*, and output), we introduce two trainable low-rank matrices $A\in R^{r\times d_{\text{in}}}$ and $B\in R^{d_{\text{out}}\times r}$. The adapted weight is formulated as:


(1) $$W^{\prime }=W+\Delta W=W+\frac{\alpha }{r}BA,$$

where *r* is the LoRA rank ($r\ll \min(d_{\text{in}},d_{\text{out}})$) and α is the LoRA scaling hyperparameter. The factor α/r follows the standard LoRA parameterization used in PEFT implementations, so the low-rank update is scaled by α/r rather than by α alone. Here, *W* is the frozen pretrained weight matrix and ΔW=(α/r)BA is the trainable low-rank update. During fine-tuning, optimization is restricted to the low-rank factors *A* and *B* and the newly introduced downstream layers, while *W* remains unchanged. This substantially reduces the number of tunable parameters (Hu *et al.* 2021) compared to full fine-tuning. We apply LoRA to the self-attention projection matrices *Q*, *K*, *V*, and the output projection in all encoder layers following (Hu *et al.* 2021), while leaving the feed-forward blocks and the embedding layer frozen. This PEFT approach retains ESM-2’s implicitly learned evolutionary and structural features while allowing LoMuS to adjust the attention dynamics for stability prediction with minimal overhead. During fine-tuning, only the LoRA parameters and subsequent new layers are updated; the vast majority of PLM’s original parameters remain fixed, avoiding overfitting and reducing GPU memory requirements.

### 2.4 Multi-representation of a protein molecule: physicochemical and basis features

LoMuS incorporates local physicochemical properties and global sequence-level descriptors in addition to sequence embeddings. We derive these features from each sequence *a priori*. The AAindex1 database is used to extract the physicochemical properties of each amino acid in the sequence (Kawashima and Kanehisa 2000). This feature set consists of 553 published indices, where each index provides a numeric value for each 20 canonical amino acid, representing physicochemical properties, such as hydrophobicity, flexibility, polarizability, chemical shifts and many. We apply an average pooling across the sequence to compute physicochemical representation of the molecule, yielding a 553-dimensional vector for the sequence. These features summarize the overall tendency of the sequence with respect to a wide range of biochemical scales.

Next, we derive six global physicochemical descriptors frequently used in protein property and stability modeling (Yang *et al.* 2022, Kuang *et al.* 2024) of a protein molecule. These include:

- Sequence length (L): Number of standard amino acids in the protein.
- Molecular weight (MW): Total mass of the protein in Daltons, computed from the sequence composition.
- Isoelectric point (IP): The pH at which the protein has net zero charge, estimated from amino acid pKa values.
- Grand Average of Hydropathy (GRAVY): The mean hydropathy value of the sequence, indicating overall hydrophobicity or hydrophilicity.
- Aliphatic index (AI): A measure of the volume of aliphatic side chains (Ala, Val, Ile, Leu) in the protein, which is empirically correlated with thermostability.
- Instability index (II): An empirical measure of in vitro stability, where a value <40 predicts a stable protein and >40 predicts an unstable protein.

These six features are derived directly from the raw amino acid sequence using Biopython’s ProteinAnalysis toolkit, implemented in line with ExPASy ProtParam conventions (Gasteiger *et al.* 2005, Cock *et al.* 2009). Finally, we have the physicochemical representation of the protein molecule by concatenating all features as in Equation 2.


(2) $$\begin{array}{c} f_{\text{phys}}=[\mu_{{\text{AAindex}}_{1}},\ldots ,\mu_{{\text{AAindex}}_{553}},L,\text{MW},\text{IP},\\ \text{GRAVY},\text{AI},\text{II}]\in R^{559}. \end{array}$$

### 2.5 LoMuS fusion architecture

After extracting the LoRA enabled residue contextual embeddings from the PLM and the global physicochemical feature vector, LoMuS employs a fusion network to predict the protein stability score. The core components of the network are discussed in the following subsections.

#### 2.5.1 Protein representation generation from amino acid level embeddings

To summarize residue-level embeddings into a fixed-length protein representation, we employ a standard attention pooling mechanism commonly used in sequence representation learning. This component serves as a lightweight and effective alternative to average or CLS-token pooling for aggregating residue-level embeddings into a sequence-level representation.

We apply an attention pooling operation on the residues to compute the protein level representation. Let $H=(h_{1},\ldots ,h_{L})$ denotes the residue embeddings extracted from the last PLM layer, where *L* is the input sequence length. The relevance score for residue *i* is determined by a learnable linear projection, $W_{\text{attn}}$, layer followed by a tanh nonlinearity, and a learned context vector *u* as in Equation 3:


(3) $$e_{i}=u^{T}\tanh\left(W_{\text{attn}}h_{i}\right).$$

Subsequently, attention weights $\alpha_{i}$ are obtained by masking padded positions with a large negative value and normalizing the scores across residues using a softmax function, as shown in Equation 4:


(4) $$\alpha_{i}=\frac{\;\text{exp}(e_{i})}{\sum_{j=1}^{L}\;\text{exp}\;(e_{j})}.$$

The sequence-level representation is a weighted average of the residue embeddings. If the weights concentrate on a few positions, the representation emphasizes those key residues.


(5) $$h_{\text{lemb}}=\sum_{i=1}^{L}\alpha_{i}h_{i}.$$

The physicochemical vector $f_{\text{phys}}\in R^{559}$ is mapped into the same latent space as the PLM embedding dimension via a multi-layer perceptron $g_{w}(\cdot )$ with nonlinearity ReLU activation function and dropout (rate = 0.1):


(6) $$h_{\text{phys}}=g_{w}(f_{\text{phys}})\in R^{d},$$

where *d* is the PLM hidden size (i.e. d=1280 for ESM-2). $g_{w}(\cdot )$ aligns scale and geometry of physicochemical features within the PLM space, stabilizing fusion and allowing richer interactions in the downstream tasks.

#### 2.5.2 Fusion of multi-representation features

We concatenate the sequence level LoRA enabled PLM embedding ($h_{\text{lemb}}$) and projected physicochemical features ($h_{\text{phys}}$) to form combined embedding as in Equation 7.


(7) $$z=[h_{\text{lemb}};h_{\text{phys}}]\in R^{2d},$$

where [*x; y*] denotes vector concatenation. We adopt concatenation as a simple and robust fusion operator that preserves the full information content of each modality before the regression head, while keeping the fusion mechanism parameter-light. This is desirable in our setting because the PLM backbone is largely frozen and only low-rank adapters and lightweight heads are trained; therefore, a low-complexity fusion strategy helps reduce overfitting risk and allows subsequent layers to learn task-specific cross-feature interactions implicitly. The fused vector *z* captures both sequence-derived patterns from the LoRA-PLM and global physicochemical properties.

#### 2.5.3 Loss/objective function

The model deploys a regression head to estimate the stability score $\hat{y}$ given the fused sequence-level vector representation *z*. We train the model end-to-end by minimizing the mean-squared error (MSE) between predictions $\hat{y}$ and ground truth labels *y*. For a mini-batch of *N* samples, the loss can be defined as:


(8) $$L_{\text{MSE}}=\frac{1}{N}\sum_{i=1}^{N}{\left({\hat{y}}_{i}-y_{i}\right)}^{2},$$

which provides a smooth regression objective to drive learning.

## 3 Datasets

To systematically assess the proposed model architecture, we utilize different publicly available datasets from large-scale to out-of-distribution test sets. We also evaluate our model for per-protein mutational scanning usecases to support rational protein engineering. In this section, we discuss each dataset with their uniqueness and highlight our preparation steps.

### 3.1 Tsuboyama mega-scale folding-stability dataset

We use the cDNA-display proteolysis dataset from Tsuboyama *et al.* (2023). It compiled and reported the absolute folding free energy ΔG for approximately $7.7\times 10^{5}$ sequences, largest publicly available protein stability dataset to the best of our knowledge, under uniform conditions near pH 7.4 and 298K. These proteins span across 331 natural and 148 *de novo* domains. Proteolysis is carried out with trypsin and chymotrypsin, and a Bayesian kinetic model estimates each sequence’s $K_{50}$ and converts it to ΔG. Quality filters remove sequences with unreliable dynamic range or evidence of folded-state cleavage, yielding a large and internally consistent corpus of ΔG measurements for stability modeling (Tsuboyama *et al.* 2023).

**Figure 2:**
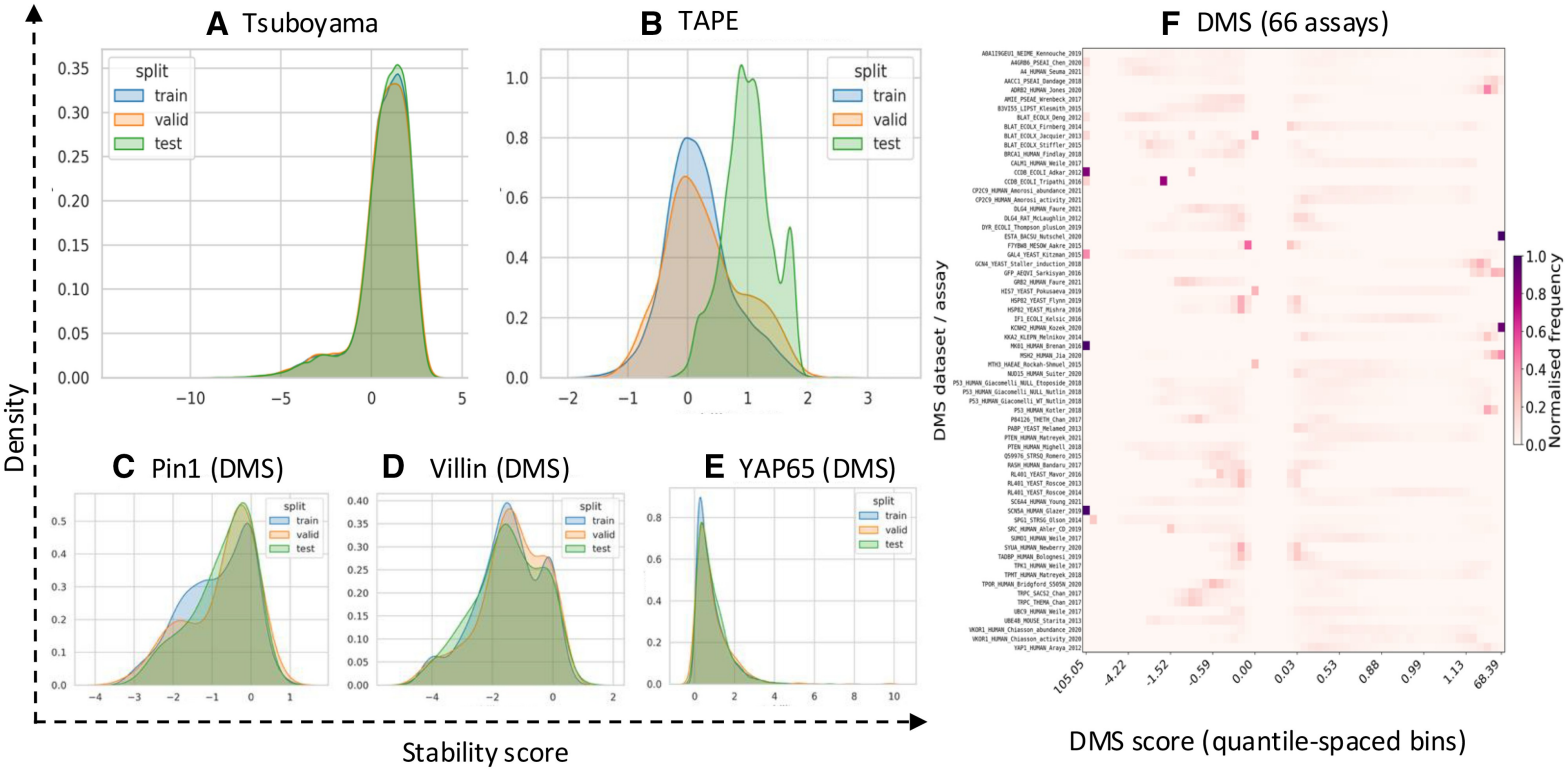
Protein folding stability label distributions by train, validation and test splits across various benchmarks. (A) Tsuboyama ΔG mega-scale folding stability dataset (Tsuboyama *et al*. 2023). (B) TAPE stability benchmark (Rao *et al*. 2019) exhibits a deliberate distribution shift, with the test set centered at a higher mean and lower variance than train and validation, emphasizing the challenge of generalization. (C, D and E) Per-protein deep mutational scanning (DMS) datasets for Pin1, Villin, and YAP65 WW (from left to right). (F) Normalized DMS score distributions across all 66 assays, displayed using quantile-spaced bins. Each row corresponds to one assay; color intensity reflects normalized frequency within each bin.

We adopt a wild-type domain aware splitting protocol for training and testing of our proposed model commonly used in proteomics research following ESMtherm (Chu *et al.* 2024). Contrary to the random mechanism, domain-based splitting considers keeping all mutant- and wild-type sequences into the same partition. This indicates that the entire domains are held out for testing and validation. Finally, the train, validation, and test sets contain 379 577, 47 414, and 100 794 sequences, respectively. Summary statistics for different partitions are reported in Table S1. We keep ΔG as a continuous target without binarization. Fig. 2(A) demonstrates the stability value distributions for all splits. Overall, this dataset evaluates the generalization capacity of the model to novel domains rather than additional mutants from the same training domains.

### 3.2 TAPE stability benchmark

Next, we evaluate our model’s generalization capability when applied to the stability values situated outside the training distribution. We adopted a stability benchmark dataset from TAPE (Rao *et al.* 2019) where the underlying experimental measurements are obtained from the large scale protein design and stability study by Rocklin *et al.* (2017). The train and validation come from four rounds of experimental measurements across many candidate proteins, while the test set is built from seventeen Hamming-1 neighborhoods centered on promising proteins identified during those rounds (Rao *et al.* 2019). The test examples stay near optimized scaffolds. This introduces a conscious label-distribution shift in the test set relative to the training and validation set. Fig. 2(B) shows the distribution shift in stability label statistics, where the test set shows a higher mean (1.002) and lower variance (0.409) than train (0.179±0.566) and validation (0.279±0.656) sets (Table S2). Since the test sequences are close mutational neighbors of top candidates, the naive finetuning over the splits emphasizes learning local landscape around optimized parents instead of learning optimal solution, thus encourages overfitting. This design choice ensures that the optimal model must learn most salient and relevant features to molecular stability.

### 3.3 Deep mutational scanning (DMS) (per-protein)

LoMuS is evaluated on two settings of per-protein DMS datasets: (i) the same collection of assays used by ProMEP (Cheng *et al.* 2024), drawn from the ProteinGym benchmark (Notin *et al.* 2023): 66 DMS datasets covering 53 distinct proteins from prokaryotes, humans, and other eukaryotes, and (ii) a targeted per-protein evaluation on the three natural-protein stability datasets used in the UniRep benchmark (Alley *et al.* 2019): the human YAP65 WW domain, the Villin headpiece, and Pin1. These two settings serve a distinct purpose. While large-scale DMS evaluation covers a wide range of proteins to assess generalization, the per-protein protocol allows a direct, controlled comparison against UniRep on the exact datasets and splits it was validated on, providing a meaningful baseline reference grounded in prior published work.

We follow UniRep’s protocol (Alley *et al.* 2019) and generate 80/10/10 train/validation/test splits for each protein using the same fixed random seed as in UniRep (Alley *et al.* 2019), holding out the test set entirely until final evaluation. Label distributions for each protein are reported in Fig. 2(C, D, E). We observe different skewness in the label distributions across proteins, which ensures a consistent comparison to the UniRep baseline and a fully independent test for each protein.

Each protein is treated as an independent task through the same pipeline. We parse the provided sequences and their experimental stability scores (numeric labels), sanitize sequences to the standard 20 amino acids, and compute the per-sequence physicochemical feature vector. Z-score label normalization is applied per protein, fit on the training split and applied to the validation and test splits, to standardize the stability scale across proteins. Summary statistics for the three protein datasets are provided in Table S3.

For large-scale DMS analysis from ProteinGym, following ProMEP, the 19 viral proteins are excluded to avoid biases from pre-training corpora. The assays differ substantially in readout (binding, catalytic activity, fluorescence, growth-based fitness, thermal stability, etc.), sequence length, and number of variants, which makes this collection a stringent per-protein generalization test. We note that ProMEP performs *zero-shot* mutation-effect prediction by combining sequence with predicted structural context from ESMFold, and therefore uses each assay entirely as a held-out test set. In contrast, LoMuS is a supervised, sequence-only model and does not consume any structural input. We therefore evaluate in the supervised per-protein regime used for the original DMS datasets in UniRep (Alley *et al.* 2019): for each of the 66 assays we generate an 80/10/10 train/validation/test split using the same fixed random seed, and the test partition is held out entirely until the final evaluation.

Figure 2(F) shows the normalized score distributions across all 66 assays using quantile-spaced bins. The heatmap reveals substantial heterogeneity in both score range and shape across assays: most datasets are centered near zero with moderate spread, while a small number of assays, such as CCDB_ECOLI_Adkar_2012 and GAL4_YEAST_Kitzman_2015, exhibit heavy left tails with extreme negative scores, reflecting assay-specific readout scales. A subset of datasets, including ESTA_BACSU_Nutschel_2020 and KCNH2_HUMAN_Kozek_2020, show distributions shifted toward positive values. This cross-assay variability motivates the per-assay z-score normalization applied during training. Aggregate summary statistics across all 66 assays are reported in Table S4.

## 4 Training details

We train LoMuS end-to-end using the AdamW optimizer, keeping the pretrained backbone weights fixed and updating only the LoRA adapters. To account for the differing roles and sizes of the components, we use two learning rates: a higher rate for the PLM branch, namely the LoRA-adapted attention projections, and a lower rate for the newly initialized fusion and regressor layers. This setup encourages the backbone’s attention projections to adapt meaningfully to the task while keeping the smaller fusion head conservative to avoid overfitting to the handcrafted features. In our experiment, we found that setting the PLM+LoRA learning rate larger (for example, 5e−4) than the fusion and regressor learning rate (for example, 1e−4) works well.

We also employ a short linear warm-up schedule at the start of training, where the learning rates ramp from 0 to their targets over the first few epochs or early percentage of steps, to stabilize optimization under the mixed frozen and adapter regime, and we apply dropout at 0.1 in the projection and pooling components. After warm-up, we either hold the rates constant or decay them slightly based on validation performance.

To resolve overfitting, we apply regularization during the training. We apply gradient clipping (clamping the gradient norm to a fixed threshold, with max L2 norm = 1.0) to avoid any outlier updates that could destabilize the LoRA weights or the MLPs. We train for a fixed number of epochs, evaluating on a validation set at regular intervals. For each benchmark, we use the train/validation/test split protocol specified in its corresponding dataset section, and the test split is kept fully held out during model selection and hyperparameter tuning. Model checkpoints are saved, and early stopping and model selection are triggered based on validation set performance (Spearman’s rank correlation), selecting the checkpoint with the highest validation Spearman’s ρ. This selection criterion aligns with the goal of getting the relative stability ranking correct, which is often important in protein engineering. MSE on the validation set is also monitored to ensure the model is not overfitting.

Once training is complete, the final model, which combines the LoRA-adapted PLM weights with the trained MLPs, is used to predict the dataset-provided target scores for the held-out test proteins. The physicochemical feature scaler fitted on the training data is applied to normalize new sequences’ features, and tokenization is done with the same PLM vocabulary to ensure consistency. The result is a single scalar prediction for each sequence, corresponding to the target score defined by the dataset, produced by a model that combines sequence-driven representations with global physicochemical knowledge. This multi-representation approach allows LoMuS to leverage the strengths of deep language models and domain-specific features, leading to improved stability prediction performance as demonstrated in the Results section.

We obtain ESM-2 from the HuggingFace Transformers library (https://huggingface.co/facebook/esm2_t33_650M_UR50D) (Lin *et al.* 2023) (ensuring consistent tokenization and model weights). Each input amino acid sequence is first tokenized with ESM-2’s tokenizer, which adds special tokens (start <cls> and end <eos>) and maps characters to token IDs. Each tokenized sequence is then fed into the ESM-2 encoder to produce a contextual embedding for each residue (plus the special tokens). Given an input amino acid sequence of length *L*, tokenization yields L+2 tokens after adding <cls> and <eos>. The ESM-2 encoder (hidden size d=1280) outputs hidden states $H\in R^{(L+2)\times d}$. For downstream pooling we discard the special tokens and retain residue embeddings only, forming $X\in R^{L\times d}$.

**Figure 3:**
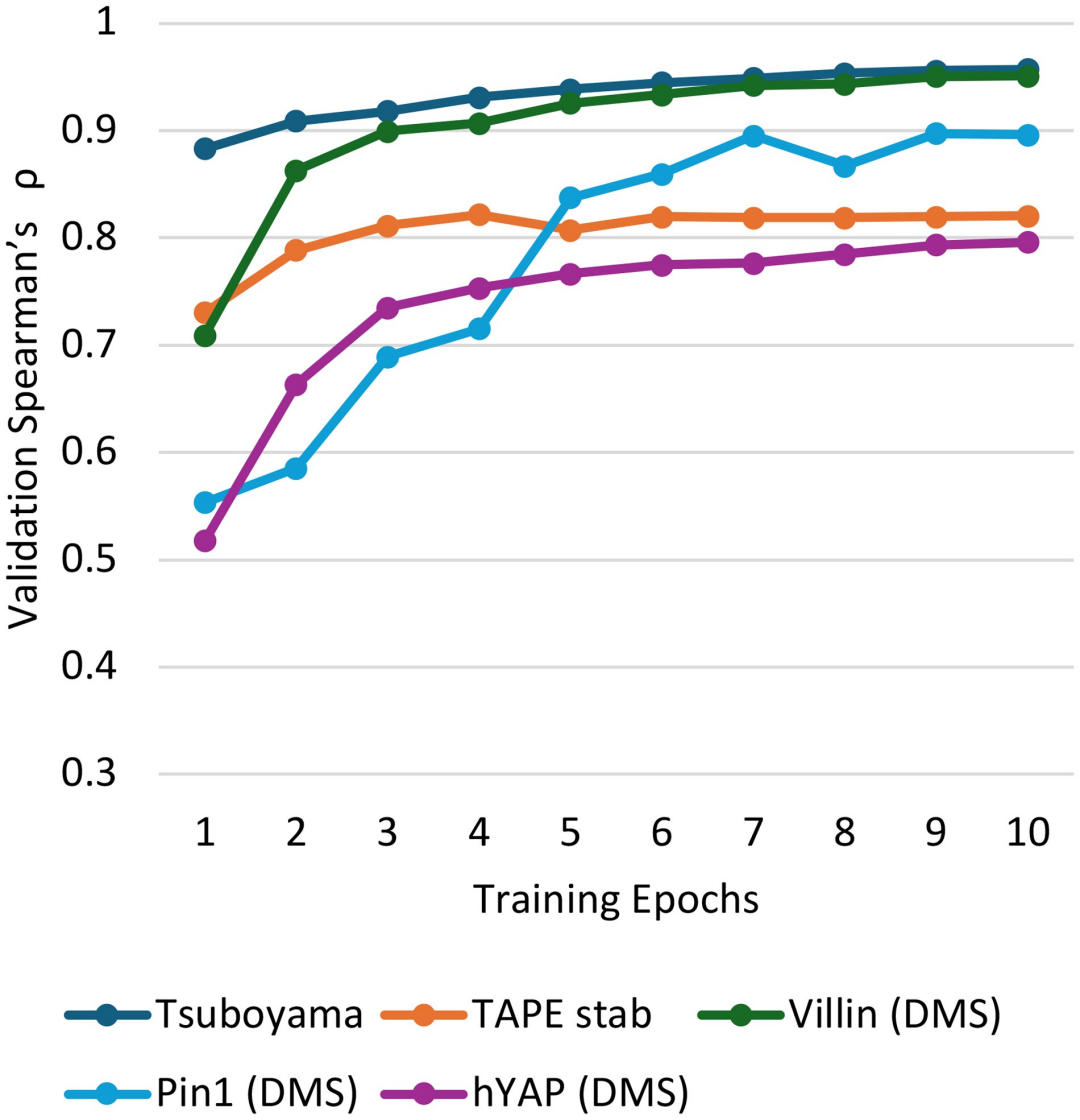
Evolution of validation Spearman’s ρ across training epochs on various benchmark datasets.

In Fig. 3, we report the validation rank correlation (Spearman’s ρ) as a function of training epoch for the main benchmark datasets discussed in Section 3. The broader 66-assay ProteinGym DMS benchmark used for the ProMEP comparison is not included in this training-curve figure because it spans many per-protein assays, which would make the plot difficult to interpret. Across the reported benchmarks, the curves show rapid gains in the first few epochs followed by a shallow plateau, indicating early convergence under a fixed training configuration.

## 5 Results and discussion

In this section, we analyze the efficacy of our proposed model compared with state-of-the-art benchmark approaches. Our results show that LoMuS’ multi-representation strategy yields substantial gains on protein stability prediction benchmarks and provides competitive performance on broader per-protein DMS assays with heterogeneous mutation-effect readouts.

### 5.1 Evaluation on held-out domains

**Figure 4:**
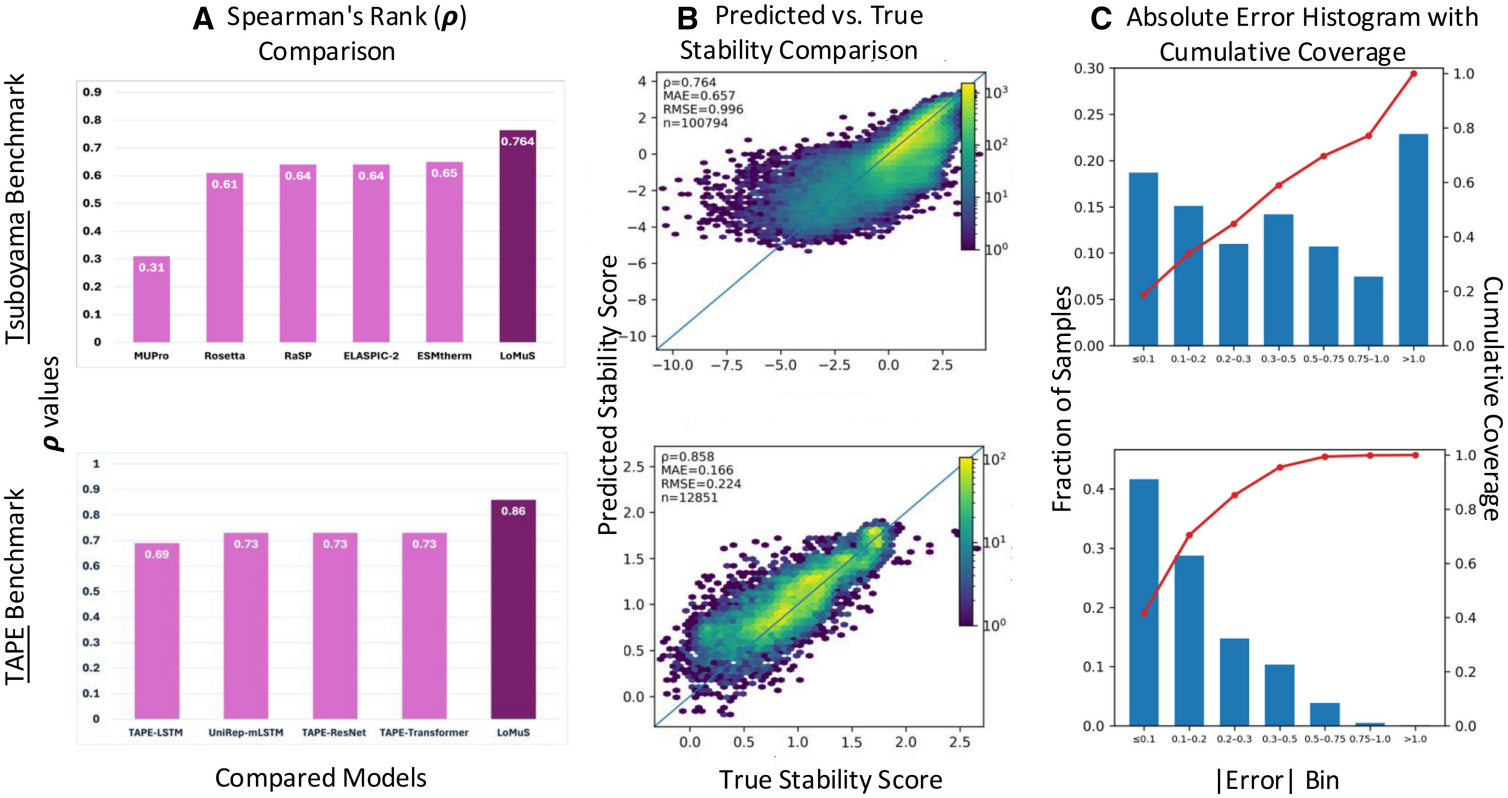
The figure highlights results across two benchmark datasets Tsuboyama (Tsuboyama *et al*. 2023) (top-panel) and TAPE (Rao *et al*. 2019) (bottom-panel). (A) Spearman’s rank correlation comparison across different models. (B) Predicted vs. ground truth stability scores comparison with Spearman’s rank correlation (ρ), mean absolute error (MAE), root mean-squared error (RMSE) of *n* test sequences. (C) Absolute error histogram with cumulative coverage across multiple bins. This demonstrate the superiority and quality of our proposed approach across held-out domains settings and out-of-distribution generalization.

We evaluate our model under a standard domain-heldout setting on out-of-distribution protein domains. To this purpose, we utilize a domain-aware splitting protocol in the Tsuboyama (Tsuboyama *et al.* 2023) mega-scale folding-stability dataset. The measurements in the dataset represent short-domain ΔG under uniform assay conditions, and the splitting protocol confirms no overlapping proteins in the train-test splits belonging to the same domain. As shown in Fig. 4(A, top panel), LoMuS achieves the best performance on the Tsuboyama benchmark, reaching a Spearman rank correlation of ρ=0.764. This exceeds the reported performance of ESMtherm (Chu *et al.* 2024), a strong sequence-only supervised fine-tuning baseline, which achieves ρ=0.65, corresponding to an absolute improvement of 0.114 in Spearman’s ρ over ESMtherm. LoMuS also outperforms other established baselines shown in the comparison, including ELASPIC-2 (Strokach *et al.* 2021) (ρ=0.64), RaSP (Blaabjerg *et al.* 2023) (ρ=0.64), Rosetta (Kellogg *et al.* 2011) (ρ=0.61), and MUPro (Cheng *et al.* 2006) (ρ=0.31). These comparisons indicate that LoMuS improves over both classical stability-prediction approaches and more recent learning-based methods on this held-out domain setting.

We note that ESMtherm is a sequence-only supervised fine-tuning baseline. LoMuS likewise operates in a sequence-only setting, since the physicochemical descriptors used by our model are computed deterministically from the same primary sequence and do not require any additional structural or experimental inputs.

Next, we visualize and compare the achieved performance using the parity plot containing 100 794 test sequences. Fig. 4(B, top panel) highlights the quality of the prediction scores and reports a mean absolute error (MAE) and root mean-squared error (RMSE) of 0.657 and 0.996, respectively. Further analysis of the errors made by the model, shown in Fig. 4(C, top panel), indicates that the absolute difference between the predicted and ground-truth stability scores exceeds 1.0 for only a small fraction of test samples. Overall, our proposed model sets a new baseline in the mega-scale protein folding stability prediction benchmark.

### 5.2 Evaluation across out-of-distribution stability labels

In our second evaluation setting, we aim to find LoMuS’s effectiveness in predicting out-of-distribution protein stability scores. We utilize TAPE (Rao *et al.* 2019) stability benchmark datasets and the same splits reported in the paper, ensuring a fair comparison and no-data leakage. LoMuS outperformed TAPE benchmark models by an absolute gain of 0.13 in Spearman’s rank correlation metric (ρ), summarized in Fig. 4(A, bottom-panel). TAPE reports performance scores for models with and without self-supervised pretraining in various settings. In the original benchmark, the best pretrained Transformer and ResNet both achieved ρ of 0.73 on the independent test set, while the pretrained LSTM reaches 0.69. Another benchmark, UniRep (mLSTM) (Alley *et al.* 2019), is in the same ballpark (ρ=0.73). LoMuS obtains Spearman’s ρ of 0.86 on the TAPE stability test split, Fig. 4(A, bottom-panel), a substantial improvement over the widely cited 0.73 baseline. This closes a large portion of the remaining headroom on this task and suggests that fusing lightweight physicochemical descriptors with a LoRA adapted PLM backbone provides a complementary signal beyond sequence-only naive fine-tuning. Further analysis utilizing the parity plot and absolute-error distribution in Fig. 4(B, bottom-panel) and Fig. 4(C, bottom-panel), respectively, demonstrates the quality of the predicted stability scores across 12 851 test sequences. In particular, roughly 70% of the test samples have an absolute error below 0.2, and only a small fraction show larger errors above 0.5. Overall, LoMuS performs well under the evaluated domain-heldout and label-shift settings, within these experimental stability benchmarks.

### 5.3 Ablation study: model architecture design

To understand the robustness of our model architecture and the contributions of the multiple protein representations, specifically physicochemical properties, we performed a systematic ablation study. Table 1 compares the Spearman’s rank correlation (ρ) for different choices of the model, such as physicochemical features only mode, naively finetuned ESM-2 backbone, and systematically incorporating LoRA with features. On the Tsuboyama dataset, a fully fine-tuned ESM-2 model provides a strong sequence-only baseline, reaching a Spearman correlation of ρ = 0.67, which is close to the reported performance of ESMtherm on the same dataset. Adding the features on top of ESM-2 produces a modest gain to ρ=0.69, while using only LoRA on top of ESM-2 yields a larger improvement to 0.72. The best performance is obtained when both components are used together, with ESM2+LoRA+features reaching ρ=0.764 on Tsuboyama, an absolute increase of 0.094 over the ESM-2 baseline. We perform this ablation on the Tsuboyama ΔG dataset because it is the largest dataset considered in this work, which makes it more reliable for building a robust architecture. Overall, the pattern suggests that the features and LoRA provide complementary information and that the full LoMuS configuration is needed to fully leverage the multiple sequence-derived representations.

**Table 1:** Ablation results on the Tsuboyama and TAPE stability dataset.

Data	ESM-2 finetuned	ESM-2 + features	ESM-2 + LoRA	LoMuS
Tsuboyama	0.67	0.69	0.72	**0.764**
TAPE	0.71	0.75	**0.85**	**0.86**

The scores correspond to the Spearsman’s ρ metric.

Bold font values indicate best-performing scores.

We also perform the same ablation study on the TAPE stability dataset, results in Table 1. Starting from the ESM-2 finetuned baseline (ρ=0.71), adding only the global physicochemical features increases performance to 0.75. Introducing LoRA gives a much larger gain, with ESM-2 + LoRA reaching ρ=0.85. With the updated results, the full multi-representation variant ESM-2 + features + LoRA achieves the best performance (ρ=0.86), indicating that the feature branch provides an additional complementary improvement when combined with LoRA on TAPE. While this gain over ESM-2 + LoRA is modest, it is consistent with the idea that the physicochemical features can add signal beyond the sequence representation once the backbone is efficiently adapted. However, the TAPE stability benchmark has a known distribution shift between its training and test sets, and it is also much smaller than the Tsuboyama ΔG dataset. We therefore view the Tsuboyama ablation, where both features and LoRA contribute complementary gains on more than seven hundred thousand variants, as the primary and more reliable piece of evidence. The TAPE ablation mainly reinforces that LoRA is a key driver of performance, while the benefit of the physicochemical features can depend on dataset size and distribution.

### 5.4 Runtime analysis of ablation variants

To complement the accuracy-based ablation results, we also compared the average training runtime per epoch of the main model variants on the Tsuboyama dataset, which is the largest benchmark considered in this work and therefore provides the most reliable setting for efficiency analysis. As shown in Table 2, under the same hardware and training conditions, full ESM-2 finetuning required 9473 s per epoch, while ESM-2 + features required 9784 s. This suggests that adding the physicochemical feature branch on top of full finetuning introduces additional overhead from feature extraction, projection, and fusion.

**Table 2:** Runtime comparison across different model configurations on the Tsuboyama dataset.

Model	ESM-2 finetuned	ESM-2 + features	ESM-2 + LoRA	LoMuS
**Runtime**	9473	9784	7078	7907

Runtime is reported as average seconds per epoch, rounded to the nearest second.

In contrast, ESM-2 + LoRA was the fastest variant, requiring 7078 s per epoch, indicating that replacing full backbone updates with LoRA substantially reduces training cost while also improving predictive performance in our benchmark. The full LoMuS model required 7907 s per epoch, remaining much closer to the LoRA-only configuration than to full finetuning. This shows that most of the efficiency benefit is retained when the feature branch is combined with LoRA, as summarized in Table 2.

Overall, these results support the practical value of the PEFT design in LoMuS: LoRA provides the largest reduction in training runtime, while the added physicochemical branch introduces a moderate overhead that is justified by the improved predictive accuracy. Since Tsuboyama is both the largest dataset and the primary evidence in this study, we report runtime analysis only on this benchmark.

### 5.5 Experiments on LoRA configurations

To select the best LoRA configurations, such as α and *r*, we run a sweep of choices. Following prior guidance on effective LoRA configurations in related settings (Shen *et al.* 2024, Li *et al.* 2025), we fix the scaling to α=16 and compared ranks r∈{4,8,9,16,32}, track Spearman’s ρ and average runtime per epoch (Table 3) on the Tsuboyama (Tsuboyama *et al.* 2023) benchmark. Based on this trade-off, we selected r=9 and α=16 for all experiments and results reported in this study.

**Table 3:** LoRA rank sweep on the Tsuboyama benchmark with scaling hyperparameter α=16.

**Scaling (** α **)**	16	16	16	16	16
**Rank (*r*)**	4	8	9	16	32
**Spearman’s** ρ	0.742	0.749	**0.764**	0.745	0.752
**Avg. Runtime/Epoch (s)**	7864	7862	7907	7958	8221

Runtime is reported as average seconds per epoch, rounded to the nearest second.

Bold font values indicate best-performing scores.

### 5.6 Feature importance analysis with integrated gradients (IG)

To better understand how the additional physicochemical branch contributes to LoMuS predictions, we performed a post-hoc feature importance analysis using Integrated Gradients (IG) (Sundararajan *et al.* 2017). In this analysis, the pooled PLM representation $h_{\text{lemb}}$ was first computed and then held fixed, while IG was evaluated only with respect to the standardized physicochemical input vector $f_{\text{phys}}$. Following our architecture, the feature vector is projected as


$$h_{\text{phys}}=g_{w}(f_{\text{phys}}),$$

and the final prediction is obtained from the fused representation:


$$z=[h_{\text{lemb}};h_{\text{phys}}].$$

For a given feature vector *x* and baseline $x^{\prime }$, IG assigns the attribution of feature *i* as


$${\text{IG}}_{i}(x)=(x_{i}-x_{i}^{\prime })\int_{0}^{1}\frac{\partial F(x^{\prime }+\alpha (x-x^{\prime }))}{\partial x_{i}}d\alpha ,$$

where F(·) denotes the model output. In our implementation, $x^{\prime }$ was chosen as the zero vector in standardized feature space, and the path integral was approximated with a finite-step Riemann sum.

**Figure 5:**
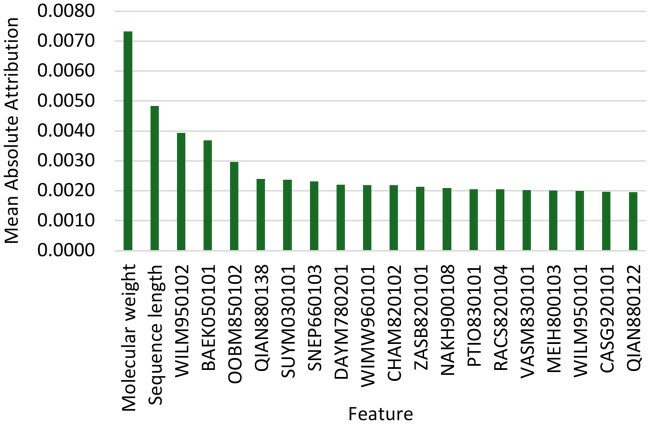
Global feature importance from Integrated Gradients on the Tsuboyama held-out test set. Features are ranked by mean absolute attribution across test samples, with attributions computed with respect to the standardized physicochemical input vector.

Figure 5 summarizes the global feature importance ranking using the mean absolute IG attribution over the test set. The results show that *molecular weight* and *sequence length* are the two most influential basis descriptors, followed by several AAindex-derived physicochemical features such as *WILM950102* and *BAEK050101*. These findings support the ablation results by showing that the handcrafted feature branch provides non-trivial complementary signal beyond the LoRA-adapted PLM embedding, while still remaining lightweight and biologically interpretable.

### 5.7 Evaluation on per-protein deep mutational scanning (DMS)

Protein engineering is a critical component in therapeutics and drug design. To address this challenge, we attempt to understand the effectiveness of our model across different individual protein settings. We evaluate LoMuS on the broader 66-assay ProteinGym DMS benchmark described in Section 3, which covers 53 distinct proteins and provides an additional test of per-protein generalization beyond the primary stability-focused benchmarks. This comparison should be interpreted with the methodological differences in mind: ProMEP performs zero-shot prediction using sequence and predicted structural context, whereas LoMuS is evaluated as a supervised sequence-only model using per-assay train/validation/test splits.

As shown in Table 4(A), LoMuS achieves a macro-average Spearman’s ρ of 0.566 across the 66 assays, compared to 0.523 for ProMEP. This corresponds to an absolute improvement of 0.043, despite LoMuS not using structural inputs (detailed result in Table S5). Since these assays include heterogeneous mutation-effect readouts beyond stability, this result suggests that the LoMuS representation can transfer to broader per-protein DMS tasks, although the primary evidence for the model remains its performance on stability-focused benchmarks.

**Table 4:** Macro-average Spearman’s rank correlation (ρ) across the 66 DMS assays, reported under each method’s corresponding evaluation setting.

**Spearman’s** ρ	ProMEP	LoMuS
(A) Macro-average	0.523	**0.566**
(B) Per-protein	**UniRep (mLSTM)**	**LoMuS**
YAP65 WW	**0.78**	0.776
Villin	0.86	**0.966**
Pin1	0.89	**0.894**

DMS per-protein results on the test set for (Spearman’s ρ) on three natural proteins.

Bold font values indicate best-performing scores.

**Figure 6:**
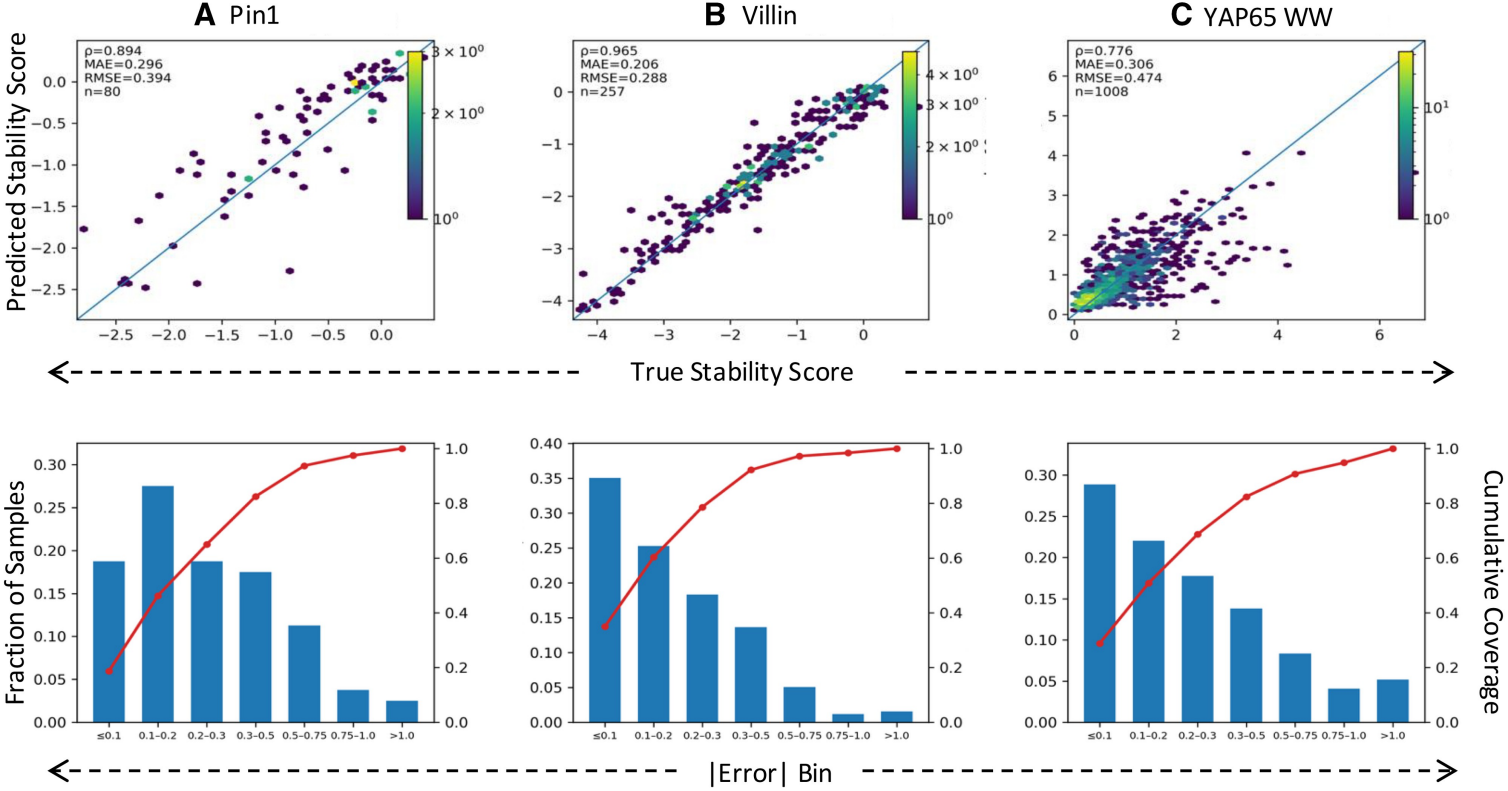
Model performance per-protein deep mutational scanning analysis for (A) Pin1, (B) Villin, and (C) YAP65 WW proteins. This demonstrate the overall quality of the model prediction scores (top panel) and low error difference (bottom-pane) across specific proteins.

Further, we utilize DMS datasets for three naturally occurring proteins, corresponding to the natural-protein DMS benchmarks evaluated in the UniRep (Alley *et al.* 2019). We compare our results with those of UniRep, a sequence-only multiplicative LSTM pretrained with large-scale self-supervision, which established a stability benchmark in DMS across multiple proteins. LoMuS achieves a Spearman’s rank correlation (ρ) of 0.894, 0.966 and 0.776 in the three test sets for Pin1, Villin and YAP65 WW, respectively (Table 4(B)). Our model outperforms UniRep or performs on par, notably obtaining an absolute performance gain of 0.11 for Villin. Figure 6 visualizes our model’s prediction quality using the parity plot (top-panel) and reports absolute prediction errors with cumulative coverage (bottom-panel) compared to the stability values of the ground-truth in the test sets across these three proteins. In all protein settings, less than 5% of the samples have an absolute error difference greater than 1.0.

Overall, our fused features captured stability determinants that a sequence-only model missed. Even when the baseline was already high, as in the case of Villin, incorporating global descriptors maintained or slightly improved performance without reducing generalization.

## 6 Conclusion and future work

Designing stable molecules is a fundamental challenge in protein engineering and therapeutics. In this work, we propose a multi-representation deep learning framework, LoMuS, to predict protein stability scores given the primary amino acid sequence as input. We evaluate LoMuS under several benchmark settings, including domain-heldout and label-shift experimental stability benchmarks, targeted per-protein stability DMS comparisons, and a broader 66-assay ProteinGym DMS evaluation across 53 proteins with heterogeneous mutation-effect readouts, demonstrating consistent performance within the evaluated scope. The core of the LoMuS’s architecture leverages a state-of-the-art PLM. It implicitly benefits the model with learned evolutionary signals and structural patterns from a large corpus of protein sequences. At the same time, the inclusion of known physicochemical descriptors provide orthogonal information compared to the embeddings. Finally, low-rank adaptation and the multi-representation fusion design help LoMuS perform robustly under the evaluated domain-heldout and label-shift settings. A reliable in silico stability predictor like LoMuS can facilitate rational design cycles in protein engineering workflows. We also anticipate that the principles of LoMuS, which combine pretrained sequence embeddings with carefully selected descriptive features in a low-rank fine-tuning framework, can be extended to other protein modeling tasks where interpretability and data efficiency are important. A systematic comparison against other PEFT alternatives (e.g. adapters, prefix tuning, IA^3^) and additional feature-fusion baselines can be future step.

With the convincing success of our proposed model, we aim to extend the multi-representation framework to incorporate richer input modalities when available. A unified stability predictor by integrating lightweight structural summaries, wet-lab or computationally derived, contact patterns, solvent exposure, and/or evolutionary context might enrich model’s confidence and improve performance. Furthermore, the physicochemical indices used in LoMuS were calibrated on folded proteins; their relevance to disordered proteins is uncertain. In practice, intrinsically disordered proteins (IDPs) or regions fed into LoMuS might be predicted as “unstable” due to high instability index or low hydrophobicity, however, this does not capture the biology of disorder. Therefore, expanding and validating the model on a wider range of protein types such as membrane proteins, multi-domain assemblies, and intrinsically disordered proteins will be another future step to consider.

In another direction, we aim to advance our proposed model to real world protein engineering practice. Instead of focusing only on stability regression, future work could engage the same multi-representation recipe on other tasks such as solubility, expression levels, binding affinity or enzyme activity, as well as on multi task setups where several properties are learned and predicted jointly. In summary, LoMuS advances the field towards predictive models of protein stability that are both highly accurate and explainable, thus supporting rational protein engineering and our understanding of sequence–stability relationships.

## Supplementary Material

btag509_Supplementary_Data

## Data Availability

No new data has been produced during this work. The data used in the model development and validation process can be found in Rao *et al.* (2019); Alley *et al.* (2019); Notin *et al.* (2023); Cheng *et al.* (2024); Tsuboyama *et al.* (2023).

## Computational requirements

Experiments are performed on a single NVIDIA A100 GPU on the GAIVI cluster, with 8 CPU cores and 32 GB RAM. Training is also feasible on a 12 GB NVIDIA GPU when using LoRA, mixed precision, and gradient accumulation. We used Python 3.10, PyTorch 2.*x* with CUDA 11.8 or newer, Hugging Face Transformers 4.40 or newer, PEFT for LoRA, SciPy, scikit-learn, NumPy, pandas, and Biopython for basis descriptors.
