# Supplementary material for: LoMuS: low-rank adaptation with sequence multi-representation improves protein stability prediction
Source: Bioinformatics. 2026 Jul 9;42(7):btag509. doi: 10.1093/bioinformatics/btag509 (PMC13391163; doi:10.1093/bioinformatics/btag509)
Supplement: btag509_Supplementary_Data [file btag509_supplementary_data.pdf]

Supplementary Information

Dataset Statistics in Various Benchmark Sources

Table S1. Summary statistics for Tsuboyama mega-scale folding-stability dataset splits.

Split	Number of Samples	Average	SD (±)
Train	379,577	0.722	1.483
Validation	47,414	0.707	1.487
Test	100,794	0.739	1.453

Table S2. Summary statistics for TAPE stability dataset splits.

Split	Number of Samples	Average	SD (±)
Train	53,614	0.179	0.566
Validation	2,512	0.279	0.656
Test	12,851	1.002	0.409

Table S3. Summary statistics for DMS per-protein dataset splits of three natural proteins.

Protein	Split	Number of Samples	Average	SD (±)
Pin1	Train	642	0.876	0.871
	Validation	80	0.756	0.864
	Test	80	0.729	0.770
Villin	Train	2,054	1.467	1.096
	Validation	257	1.368	1.090
	Test	257	1.486	1.114
YAP65	Train	8,059	0.855	0.807
	Validation	1,008	0.897	0.925
	Test	1,008	0.866	0.723

Table S4. Aggregate summary statistics for the 66 DMS assays. Label statistics are computed from the ground-truth sequence-level scores within each assay and summarized across assays.

Split	# Assays	Number of Samples	Average	SD
Train	66	1,149,612	1.3316	0.8122
Validation	66	143,674	1.2968	0.8402
Test	66	143,761	1.3209	0.8001

Detailed Results for the 66 DMS Datasets

As supporting material for the broader DMS benchmark, we provide the per-assay performance of LoMuS across the 66 Deep Mutational Scanning (DMS) datasets used in the ProteinGym-based evaluation. These assays cover diverse proteins and experimental readouts, including binding, activity, fluorescence, fitness, and stability-related measurements. Because the label scales differ substantially across assays, we report Spearman's rank correlation coefficient (ρ) on the held-out test split for each dataset. The macro-average is computed by averaging Spearman's ρ equally across the 66 assays, rather than weighting by the number of variants in each assay.

Table S5: Per-assay LoMuS performance on the 66 DMS datasets. The reported metric is Spearman's rank correlation coefficient (ρ) on the held-out test split for each assay.

**DMS assay**	**Test variants (n)**	**Spearman's ρ**
A0A1I9GEU1_NEIME_Kennouche_2019	93	0.1443
A4GRB6_PSEAI_Chen_2020	501	0.6979
A4_HUMAN_Seuma_2021	1,449	0.6728
AACC1_PSEAI_Dandage_2018	181	0.2312
ADRB2_HUMAN_Jones_2020	780	0.5547
AMIE_PSEAE_Wrenbeck_2017	624	0.5438
B3VI55_LIPST_Klesmith_2015	789	0.6481
BLAT_ECOLX_Deng_2012	501	0.8150
BLAT_ECOLX_Firnberg_2014	479	0.8841
BLAT_ECOLX_Jacquier_2013	100	0.3881
BLAT_ECOLX_Stiffler_2015	501	0.9041
BRCA1_HUMAN_Findlay_2018	58	0.4110
CALM1_HUMAN_Weile_2017	182	0.1797
CCDB_ECOLI_Adkar_2012	119	0.6958
CCDB_ECOLI_Tripathi_2016	167	0.6058
CP2C9_HUMAN_Amorosi_abundance_2021	637	0.4907
CP2C9_HUMAN_Amorosi_activity_2021	615	0.3595
DLG4_HUMAN_Faure_2021	699	0.8367
DLG4_RAT_McLaughlin_2012	159	0.4308
DYR_ECOLI_Thompson_plusLon_2019	237	0.6687
ESTA_BACSU_Nutschel_2020	218	0.2141
F7YBW8_MESOW_Aakre_2015	920	0.5502
GAL4_YEAST_Kitzman_2015	120	0.3933
GCN4_YEAST_Staller_induction_2018	265	0.5619
GFP_AEQVI_Sarkisyan_2016	5,172	0.8712
GRB2_HUMAN_Faure_2021	6,338	0.8812
HIS7_YEAST_Pokusaeva_2019	49,615	0.7405
HSP82_YEAST_Flynn_2019	1,320	0.2696
HSP82_YEAST_Mishra_2016	433	0.5734
IF1_ECOLI_Kelsic_2016	138	0.7241
KCNH2_HUMAN_Kozek_2020	20	0.2752
KKA2_KLEPN_Melnikov_2014	496	0.7854
MK01_HUMAN_Brenan_2016	682	0.4840
MSH2_HUMAN_Jia_2020	1,676	0.3024
MTH3_HAEAE_Rockah-Shmuel_2015	179	0.5071
NUD15_HUMAN_Suiter_2020	285	0.7706
P53_HUMAN_Giacomelli_NULL_Etoposide_2018	748	0.3458
P53_HUMAN_Giacomelli_NULL_Nutlin_2018	748	0.3299
P53_HUMAN_Giacomelli_WT_Nutlin_2018	748	0.8614
P53_HUMAN_Kotler_2018	106	0.4422
P84126_THETH_Chan_2017	153	0.8005
PABP_YEAST_Melamed_2013	3,772	0.9340
PTEN_HUMAN_Matreyek_2021	509	0.6506
PTEN_HUMAN_Mighell_2018	726	0.5872
Q59976_STRSQ_Romero_2015	301	0.3798
RASH_HUMAN_Bandaru_2017	314	0.7991
RL401_YEAST_Mavor_2016	126	0.7430
RL401_YEAST_Roscoe_2013	120	0.8539
RL401_YEAST_Roscoe_2014	138	0.6881
SC6A4_HUMAN_Young_2021	1,159	0.3497
SCN5A_HUMAN_Glazer_2019	23	0.0059

Continued on next page

Table S5: Per-assay LoMuS performance on the 66 DMS datasets. Continued.

**DMS assay**	**Test variants (n)**	**Spearman's ρ**
SPG1_STRSG_Olson_2014	53,697	0.9656
SRC_HUMAN_Ahler_CD_2019	338	0.3327
SUMO1_HUMAN_Weile_2017	170	0.6154
SYUA_HUMAN_Newberry_2020	251	0.6611
TADBP_HUMAN_Bolognesi_2019	121	0.6795
TPK1_HUMAN_Weile_2017	319	0.3284
TPMT_HUMAN_Matreyek_2018	366	0.5570
TPOR_HUMAN_Bridgford_S505N_2020	57	0.5684
TRPC_SACS2_Chan_2017	153	0.7392
TRPC_THEMA_Chan_2017	153	0.4382
UBC9_HUMAN_Weile_2017	257	0.5273
UBE4B_MOUSE_Starita_2013	91	0.3227
VKOR1_HUMAN_Chiasson_abundance_2020	270	0.7202
VKOR1_HUMAN_Chiasson_activity_2020	71	0.2821
YAP1_HUMAN_Araya_2012	1,008	0.7566
**Macro-average**	**143,761**	**0.5656**
